# Beauty and cuteness in peripheral vision

**DOI:** 10.3389/fpsyg.2015.00566

**Published:** 2015-05-05

**Authors:** Kana Kuraguchi, Hiroshi Ashida

**Affiliations:** Department of Psychology, Graduate School of Letters, Kyoto UniversityKyoto, Japan

**Keywords:** attractiveness, peripheral vision, face, beauty, cuteness

## Abstract

Guo et al. (2011) showed that attractiveness was detectable in peripheral vision. Since there are different types of attractiveness (Rhodes, 2006), we investigated how beauty and cuteness are detected in peripheral vision with a brief presentation. Participants (*n* = 45) observed two Japanese female faces for 100 ms, then were asked to respond which face was more beautiful (or cuter). The results indicated that both beauty and cuteness were detectable in peripheral vision, but not in the same manner. Discrimination rates for judging beauty were invariant in peripheral and central vision, while discrimination rates for judging cuteness declined in peripheral vision as compared with central vision. This was not explained by lower resolution in peripheral vision. In addition, for male participants, it was more difficult to judge cuteness than beauty in peripheral vision, thus suggesting that gender differences can have a certain effect when judging cuteness. Therefore, central vision might be suitable for judging cuteness while judging beauty might not be affected by either central or peripheral vision. This might be related with the functional difference between beauty and cuteness.

## Introduction

It is well known that an attractive face captures attention (Shimojo et al., 2003; Leder et al., 2010) and Guo et al. (2011) showed that attractiveness is even detectable in peripheral vision. They also discussed that low spatial frequency information can be used for judging attractiveness. These findings have suggested that judging attractiveness is possible even though available visual information can be limited. This is related to the idea that attractiveness is important for mate selection. Attractiveness, however, has both sexual and non-sexual aspects, such as attractiveness as a potential ally and cuteness in addition to sexual attractiveness (Rhodes, 2006). For example, we can point out the mere exposure effect (Zajonc, 1968; Peskin and Newell, 2004), that self-resemblance of same gender faces increase the attractiveness (DeBruine, 2004), and that smiling increases the attractiveness rating (Reis et al., 1990). These are mentioned as social attractiveness, which leads to establishment of the relationship of trust and aid with the other person, rather than sexual attractiveness. In fact, the dominant visual field (hemisphere) differs by the type of judged attractiveness. Sexual attractiveness (for a date situation) is more related to the left visual field (right hemisphere) while non-sexual attractiveness (as a lab partner) is more related to the right visual field (left hemisphere; Franklin and Adams, 2010). The attractiveness as a lab partner in Franklin and Adams (2010) is considered to be social attractiveness. Thus, it should be investigated how the different types of attractiveness are detected in peripheral vision.

Beauty consists of averageness, symmetry, and sexual dimorphism, all of which might show the quality of one’s genes (Thornhill and Moller, 1997) and the state of one’s health (Rhodes et al., 2001). As a result, such aspects of beauty provide important information for mate selection and function as an innate component of attractiveness. Conversely, cuteness represents the attractiveness of infants (Karraker and Stern, 1990) and is related to the baby schema concept (Hildebrandt and Fitzgerald, 1979; Alley, 1981). This concept elicits caregiving behaviors, which has been proven effective even for adult faces (Keating et al., 2003). In this regard, cuteness functions as social attractiveness in interactions with other people. Accordingly, it is possible that beauty and cuteness might reveal different aspects of attractiveness (Geldart, 2010). Attractive female faces, however, possess both neonate and sexually dimorphic features (Cunningham, 1986; Pfluger et al., 2012), therefore the criteria of judging beauty and cuteness might be overlapping. Kuraguchi et al. (2015) showed that beauty and cuteness might represent different aspects of attractiveness even though similar facial features affected both judgments to some degree. Therefore, the first aim of this study is to investigate whether beauty and cuteness are distinguished through availability in peripheral vision. We also aimed to extract the difference between beauty and cuteness from participant’s natural responses without the definition of two word meanings, in order to investigate whether sexual and social attractiveness is distinguished even in the expression used in daily life.

Moreover, the effect of gender was not considered by Guo et al. (2011). While one study showed that participants can assess the attractiveness of both male and female faces in a similar manner regardless of their gender or sexual orientation (Kranz and Ishai, 2006), it has been argued that facial attractiveness provides the adaptive benefits (i.e., signals of good genes) for different-sex observers, but not for same-sex observers (Senior, 2003). It was also reported that women were more sensitive in perceiving cuteness than men (Sprengelmeyer et al., 2009). Therefore, our second aim is to examine whether there are gender differences in judging beauty and cuteness in peripheral vision.

Furthermore, Japanese people tend to confuse beauty with cuteness (Daibo, 2007). However, as mentioned earlier, these two characteristics are considered to represent different aspects of attractiveness. It was also reported that beauty and cuteness were distinguished in the North American culture (Geldart, 2010). It is possible that peripheral viewing might reveal the difference between beauty and cuteness for Japanese people. Therefore, our third aim is to investigate how Japanese people perceive beauty and cuteness in peripheral vision.

## Experiment 1

### Methods

#### Participants

The participants consisted of 45 Japanese students (18–25 years; 22 males and 23 females) with normal vision or corrected to normal vision, who were naïve to the experimental purposes and asked to view the stimulus presentation. The distance between the eyes and the display was 56 cm, and a chin rest was used to stabilize the head. We obtained written informed consent from all the participants, and each individual was paid according to the standards of Kyoto University. During the experiment, their fixations were monitored with the Tobii T120 Eye Tracker.

#### Stimuli

**FIGURE 1:**
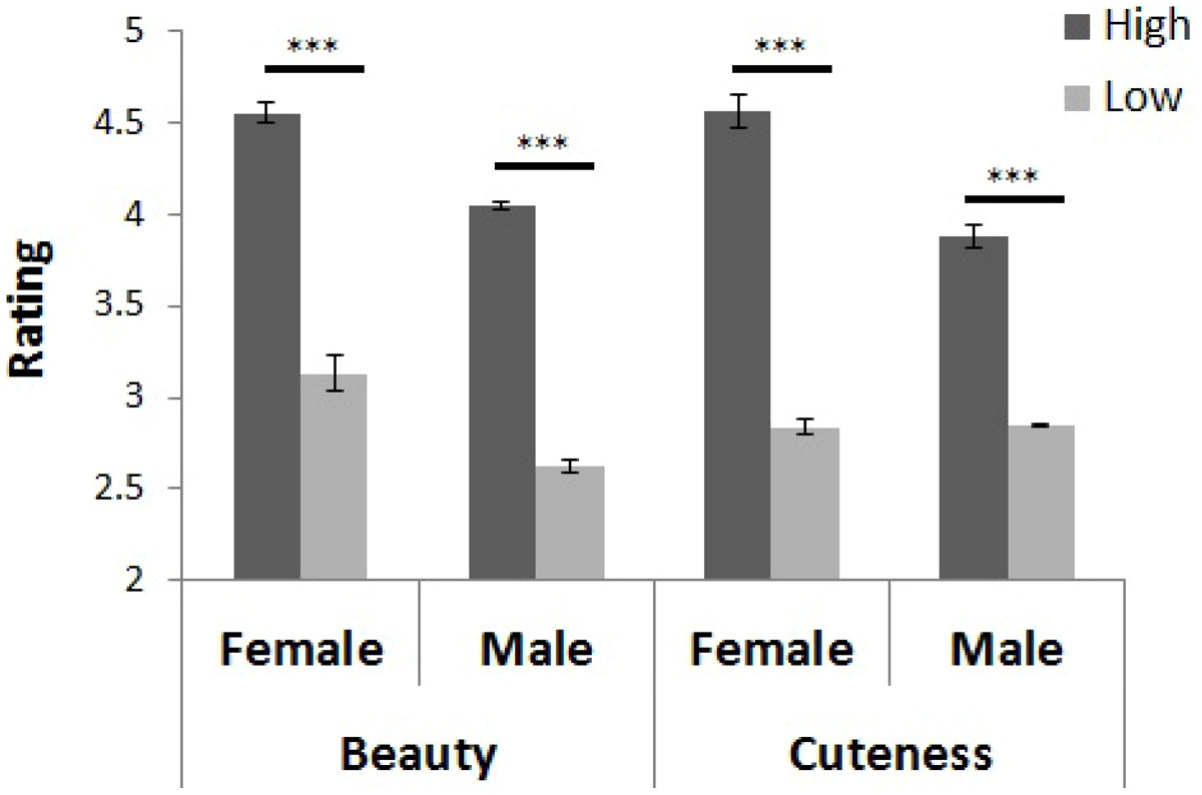
**Stimulus grouping. The deep gray shows high rated groups, and the light gray shows low rated groups**. The error bars show the SEM across participants. ***p < 0.001.

Visual stimuli were presented on a 17-inch LCD monitor (Tobii T120, 1280 × 1024 pixels, 60 Hz), and the average luminance of the stimuli was 9.94 cd/m^2^. Images of 10 Japanese female faces (18–24 years) were presented in a visual angle of 9° and by 6°. All the faces included frontal views with neutral expressions. The images were gray scale and cropped to remove external features (e.g., hair style) and they were classified into high and low groups of five faces each through a preliminary test for both beauty and cuteness in which a separate group of participants (*n* = 29) rated the images on a 6-point scale. These groups were used for the discrimination rate analysis. As **Figure 1** shows, we found significant differences in the mean rating scores of five faces between the high and low groups in both beauty [*t*(4) = 14.168, *p* < 0.001] and cuteness [*t*(4) = 12.947, *p* < 0.001]. The stimulus groups for beauty and cuteness judgments actually included exactly the same faces for female participants, and only one different face for male participants.

#### Judgment Conditions

Judgments included two conditions, beauty or cuteness, as a between-participant factor (beauty: 9 males and 10 females; cuteness: 13 males and 13 females). The participants were asked to choose the more beautiful or cuter face out of the two facial images simultaneously presented, and respond by pressing a key after the faces disappeared.

#### Procedure

**FIGURE 2:**
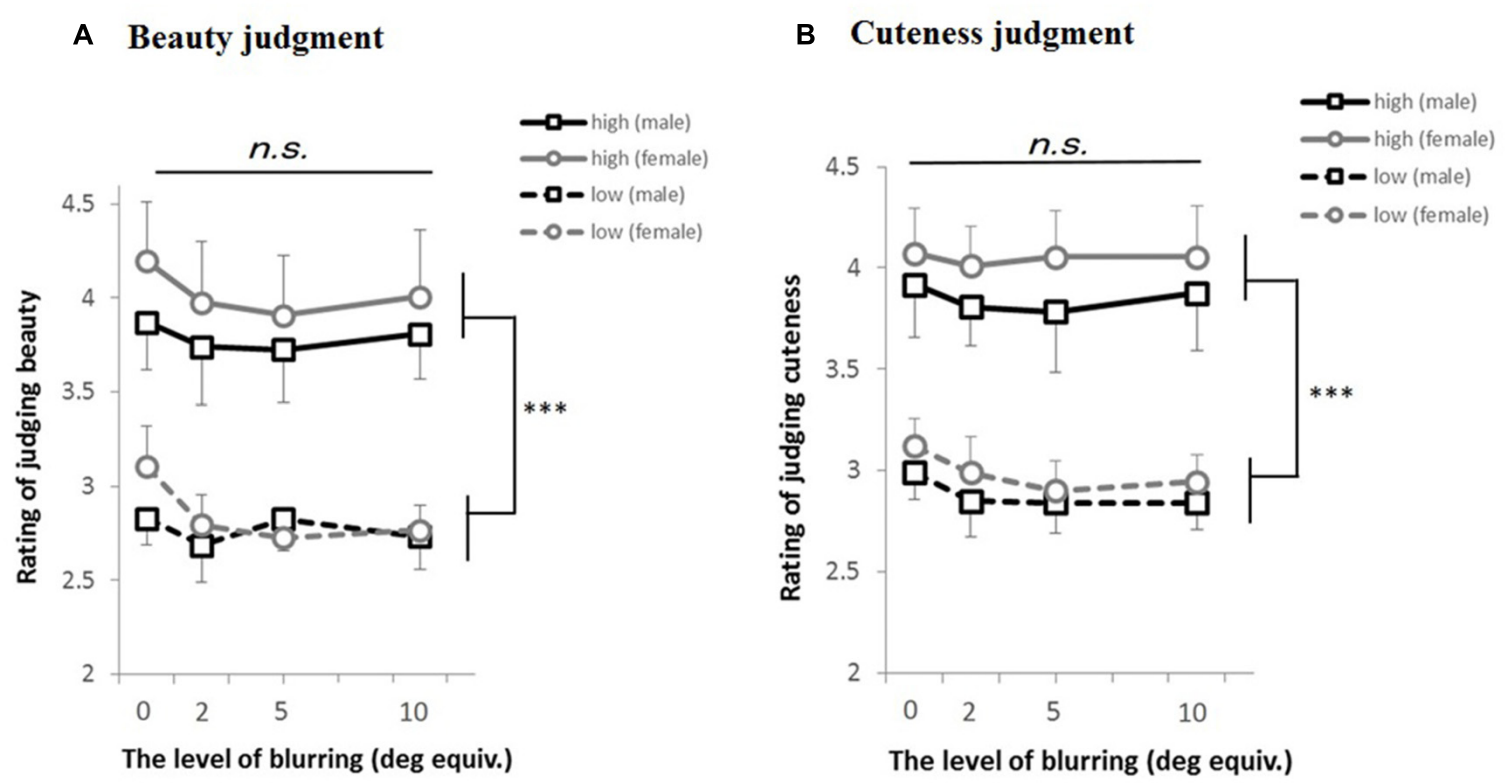
**The procedure of cuteness judgment**. We asked participants “which is more beautiful?” in case of beauty judgment.

Each trial began with a warning tone, followed by a central fixation cross shown for 1.5 s. In the same manner as Guo et al. (2011), a pair of faces was presented for 100 ms to the left and right with equal distances from the central fixation cross. The participants were asked to maintain their visual fixation and respond by pressing one of the two keys to indicate which face was more beautiful or cuter after the faces disappeared. There was no need for a quick response. **Figure 2** presents the aforementioned procedure. Pairs of faces were made by combining round-robbin (_10_C_2_ = 45 pairs), with the presented sides (left/right) counter-balanced (90 patterns in total). The data of high–low pairs were analyzed, and the other half (high–high and low–low combinations) were discarded.

The viewing eccentricity was 2°, 5°, and 10° (from the center to the inner edge of the faces), to probe foveal, parafoveal, and peripheral vision, respectively, as in Guo et al. (2011). The number of trials was 90 per eccentricity (270 in total) for each participant, tested in a random order. After this main experiment, all the participants performed a self-paced task without fixation and with unlimited presentation time. In the self-paced task, a pair of faces was presented to the left and right with equal distances (2°) from the central fixation cross, and the participants were asked to respond by pressing one of the two keys to indicate which face was more beautiful or cuter while looking at faces.

### Results

#### Consistency of Rating Beauty and Cuteness

In order to confirm the consistency for rating beauty and cuteness between the main and preliminary experiments, the facial images were ranked based on the results of the self-paced judgments by using paired comparison (Thurston’s method). In addition, the ranks of the faces were compared to those from the preliminary experiment. Significant rank correlations were found for judging both beauty and cuteness regardless of the participant’s gender (*p*s < 0.001). Accordingly, the groupings of the high and low rated for both the judgments were consistent. Furthermore, the high- and low-rated faces did not exchange with one another for both beauty and cuteness.

#### Analysis of the Discrimination Rates

We checked the participants’ fixation in stimulus presentation, and discarded all data of the participants whose fixation exceeded 1° for more than 5% of the total looking time. We therefore discarded the data from 14 participants whose fixation was unstable, and analyzed the data of 31 participants (beauty: nine males and nine females; cuteness: six males and seven females).

Discrimination rate was defined as the rate of responses that were congruent with the pre-defined high and low groups. For beauty judgment, all the discrimination rates were significantly above the chance level (50%), regardless of the participant’s gender [males: 2° *t*(9) = 3.56, *p* = 0.006, 5° *t*(9) = 3.63, *p* = 0.005, 10° *t*(9) = 4.87, *p* < 0.001; females: 2° *t*(9) = 6.09, *p* < 0.001, 5° *t*(9) = 6.98, *p* < 0.001, 10° *t*(9) = 5.54, *p* < 0.001]. For cuteness judgment, all the discrimination rates of the female participants were significantly above chance level [2° *t*(9) = 4.04, *p* < 0.001, 5° *t*(9) = 4.96, *p* < 0.001, 10° *t*(9) = 3.80, *p* = 0.004], but the discrimination rate of male participants at the eccentricity of 10 was not above-chance [2° *t*(9) = 4.92, *p* < 0.001, 5° *t*(9) = 4.64, *p* = 0.001, 10° *t*(9) = 1.55, *p* = 0.155].

**FIGURE 3:**
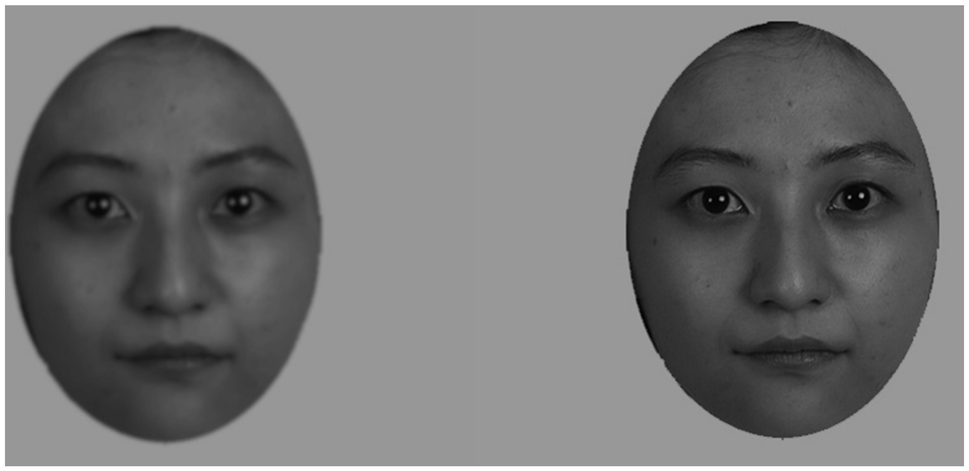
**Discrimination rates plotted against viewing eccentricity. (A)** Shows the results of judging beauty, while **(B)** shows the results of judging cuteness. The black and gray lines represent male and female participants, respectively. The error bars show the SEM across stimulus faces. ^∗∗^*p* < 0.01, *p < 0.05.

We then conducted a three-way ANOVA (2: judgment, 2: gender difference of participants, 3: eccentricity). The main effect of eccentricity [*F*(2,72) = 11.28, *p* < 0.001], the interaction between judgment and eccentricity [*F*(2,72) = 6.84, *p* = 0.001], and the interaction among judgment, gender, and eccentricity [*F*(2,72) = 4.59, *p* = 0.013] were significant. A Mendoza’s multisample sphericity test revealed that sphericity assumption was satisfied (*p* = 0.25). A simple main effect test for the interaction between judgment and eccentricity revealed the effect of eccentricity on judging cuteness [*F*(2,72) = 17.80, *p* < 0.001]. A multiple comparison test (Ryan’s method) revealed significant differences between 2° and 5°, between 2° and 10°, and between 5° and 10° (*ps* < 0.05). Simple interaction between judgment and eccentricity of the male participants was found [*F*(2,72) = 10.94, *p* < 0.001]. We also found the simple–simple main effect of judgment for the male participants in the visual angle of 10 [*F*(1,108) = 6.19, *p* = 0.014], the effect of eccentricity on judging cuteness for the male participants [*F*(2,72) = 15.71, *p* < 0.001], and the effect of eccentricity on judging cuteness for the female participants [*F*(2,72) = 4.17, *p* = 0.019]. A multiple comparison test (Ryan’s method) revealed significant differences between 2° and 10°, and between 5° and 10° for the male participants (*p*s < 0.05), and significant differences between 2° and 10° for the female participants (*p* < 0.05). No such simple effects were found for beauty judgments. These results are summarized in **Figure 3**.

**FIGURE 4:**
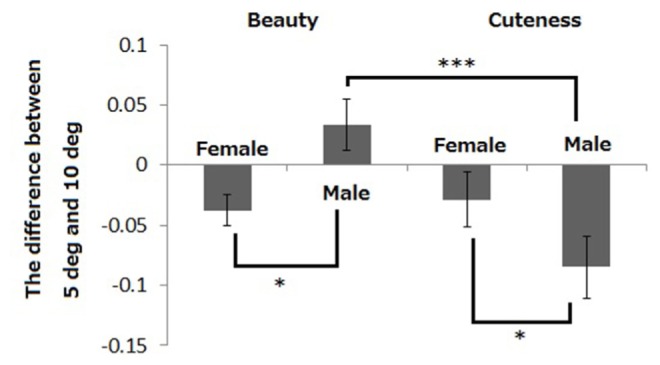
**The difference in accuracy rates between 5° and 10°**. The error bars show the SEM across stimulus faces. ***p < 0.001, *p < 0.05

For further support of the gender difference in the decline from 5° to 10°, we conducted another statistical analysis on the difference between the results of 5° and 10°. A two-way ANOVA (2: judgment type, 2: participants’ gender) revealed significant interaction [*F*(1,9) = 8.68, *p* = 0.016]. Following simple main effect analyses revealed the effect of judgments on male participants [*F*(1,18) = 13.26, *p* = 0.001], and the effect of gender differences both on judging cuteness [*F*(1,18) = 4.61, *p* = 0.045] and beauty [*F*(1,18) = 7.32, *p* = 0.014]. Gender difference is evident in judging not only cuteness but also beauty (see **Figure 4**).

### Discussion

Beauty was judged correctly in all eccentricities above the chance level, regardless of the participant’s gender. This result showed that beauty is detectable in peripheral vision, thus replicating the results of Guo et al. (2011), and beauty judgment was hardly affected by eccentricity.

However, the judgment of cuteness was affected by eccentricity. We found a significant difference between central vision (2°) and peripheral vision (10°), regardless of the participant’s gender. Judging cuteness in central vision is more accurate than in peripheral vision, even though judging cuteness is partly possible in peripheral vision. In addition, central vision could be more suitable for judging cuteness, and based on the fixation data, more participants were excluded in judging cuteness than in judging beauty, which also supports our finding that judging cuteness is difficult in peripheral vision.

Furthermore, gender difference was found in accuracy rates between 5° and 10°. In judging cuteness, the performance of males significantly declined more than that of females. In judging beauty, the performance of females declined more than that of males, while the overall decline was not significant for either gender (**Figure 3**). Significant difference was also found between beauty and cuteness in the performance of males. Accordingly, males were able to judge beauty but not cuteness in peripheral vision, while females were able to judge both beauty and cuteness. This also highlights the difference between beauty and cuteness in peripheral vision.

Then what is the cause of such a difference in central and peripheral vision? An obvious factor is blurred retinal images at the periphery. Therefore, in Experiment 2, we showed blurred images of faces in central vision that matched the perceptual blur at each eccentricity in order to investigate whether the aforementioned results can be explained solely by the blurred image.

## Experiment 2

We tested the effect of blurred faces on judging beauty or cuteness.

### Methods

#### Participants

The participants consisted of 31 Japanese students (18–36 years: 16 males, 15 females), with normal vision or corrected to normal vision, who were naïve to the experimental purposes and asked to view the stimulus presentation. No one had participated in Experiment 1. The distance between the eyes and the display was 48 cm and a chin rest was used to stabilize the head. We obtained written informed consent from all the participants, and each individual was paid according to the standards of Kyoto University.

#### Stimuli

The 10 facial images used in Experiment 1 were blurred by convolution with a 2-D Gaussian kernel of variable SD by using GNU Octave. All the faces were presented at the center of an LCD screen (Mitsubishi 23′ LCD) with a visual angle of 9° by 6° as in Experiment 1. Eye movement was not monitored. The luminance profile of the monitor was measured and was taken into account in blurring the images.

**FIGURE 5:**
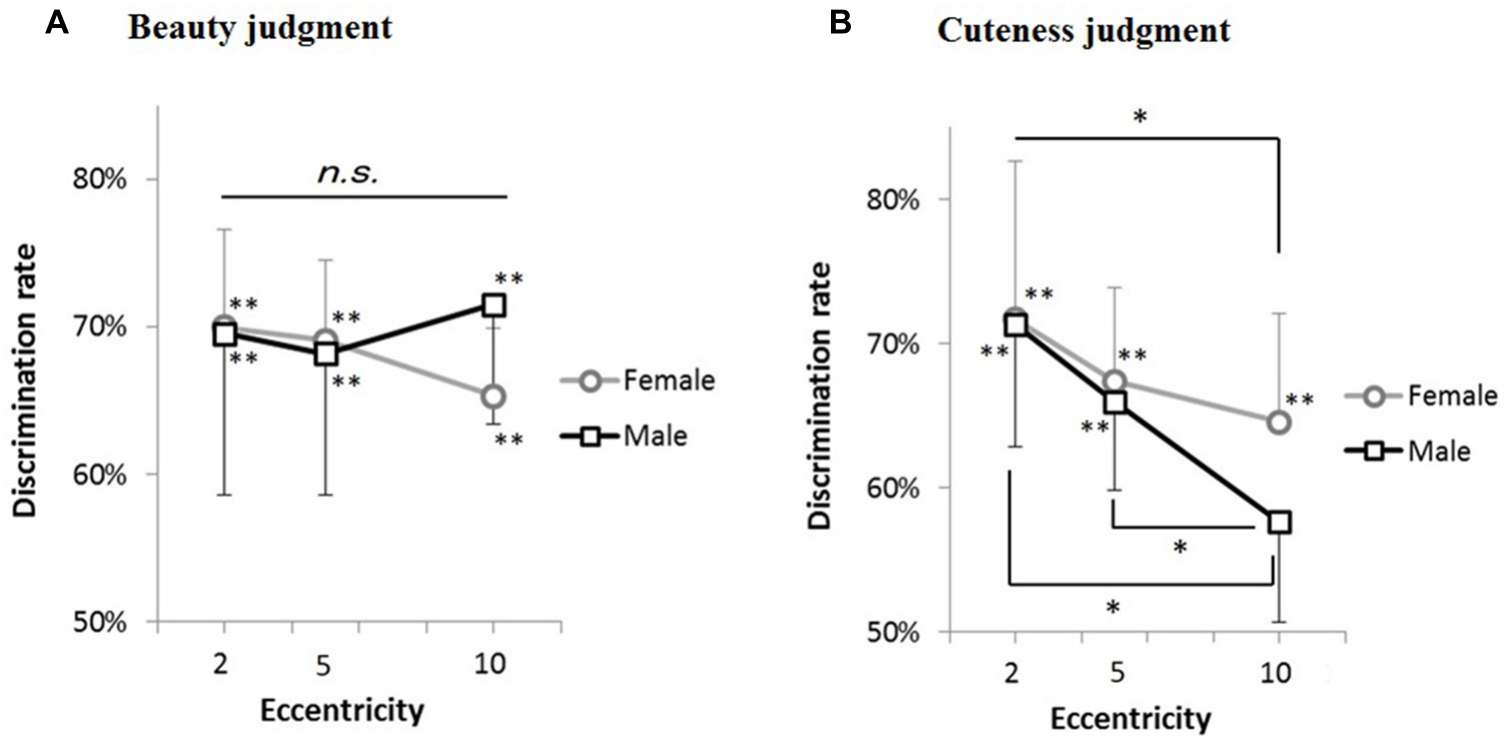
**Sample facial images. (Left)** A blurred image corresponding to the eccentricity of 10°. **(Right)** The image without blur.

First, we conducted an experiment to estimate the points of subjective equality (PSE) for blurred faces that correspond to each eccentricity. A separate group of 14 Japanese students (19–25 years: six males, eight females) participated. SuperLab 4.5 for Windows (Cedrus, Inc.,) was used to control the experiment. We presented one of the blurred images at the center of the screen and the original (not blurred) image at one of the eccentricities (2°, 5°, and 10°), either to the left or to the right, for 100 ms. Participants were asked to compare the two faces and judge if the central image appeared clearer than the peripheral one. Eight levels of blurred images were made for the two faces. Each image was repeated 10 times at the three eccentricities and at the two sides in a random order (960 trials in total). The PSEs were calculated for individual participants as the 50% level of the psychometric function that was estimated by the probit analysis, using the glm() function of R language. On average, the Gaussian half width at half maximum (HWHM) of 31.72 cycle/face-width (c/fw) corresponded to the eccentricity of 2°, 30.68 c/fw corresponded to the eccentricity of 5°, and 28.97 c/fw corresponded to the eccentricity of 10 (see **Figure 5**).

#### Judgment Conditions

Judgments were made for two conditions, beauty, or cuteness, as the factor between participants. Participants were asked to judge the beauty or cuteness on a 6-point scale, ranging 1 (e.g., not cute) to 6 (e.g., very cute), and respond by pressing a key after the faces disappeared.

#### Procedure

Each trial began with a central fixation and the participants pressed a key while viewing this fixation. A blurred face or not-blurred face was then presented at the center of screen for 100 ms. Participants were asked to fix their eyes on the face stimulus and judge beauty or cuteness on a 6-point scale presented on the display after the face disappeared, and respond by pressing one of the six keys. The number of trials was 120 trials per participant [10 images × 4 eccentricity equivalents (0°, 2°, 5°, and 10°) × 3], tested in a random order. SuperLab 4.5 for Windows (Cedrus, Inc.,) was used to control the experiment.

### Results

We conducted a three-way ANOVA [2: stimulus group (high rating/low rating), 2: participants’ gender, 4: blurring (eccentricity equivalents)] on the mean rating values of five images for beauty and cuteness ratings. In cuteness judgment, the main effect of stimulus group was significant [(*F*(1,13) = 160.473, *p* < 0.001], whereas the other effects or interactions were not significant (*p* > 0.10, see **Figure 3**). Moreover, in beauty judgment, the main effect of stimulus group was significant [*F*(1,14) = 201.763, *p* < 0.001], whereas the other effects or interactions were not significant [*p* > 0.10, see **Figure 3**].

### Discussion

**FIGURE 6:**
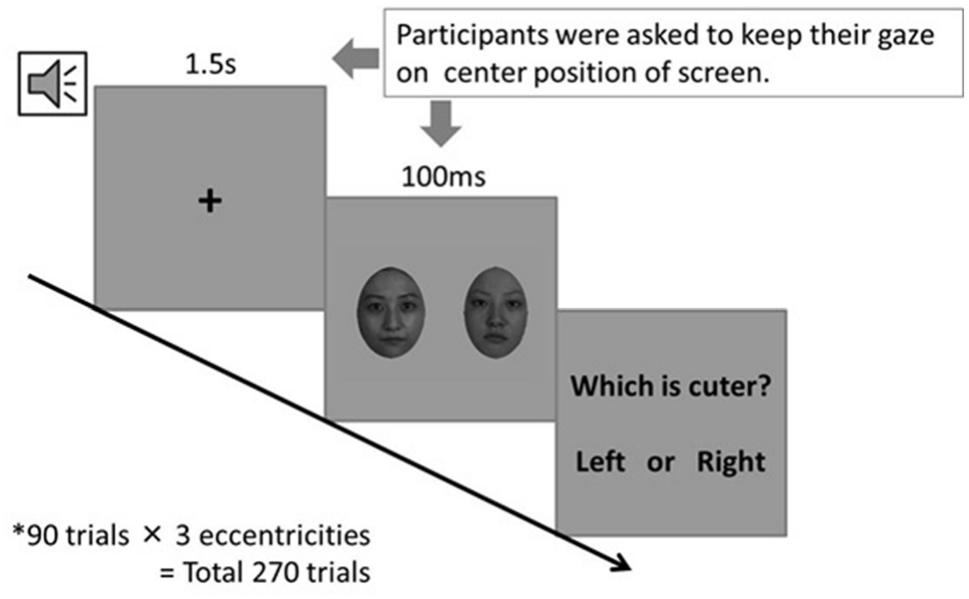
**The results of rating beauty (A)** and cuteness **(B)** in Experiment 2. The black lines represent the male participants, and the gray lines represent the female participants. The solid lines show the data of the high group used in Experiment 1, and the broken lines show the data of the low group. The error bars show the SEM across participants. ***p < 0.001.

The pattern of results in **Figure 6** is clearly distinct from those in **Figure 3**. First, the results of beauty and cuteness judgments in Experiment 2 were similar to each other, which indicate that image blur is not the primary cause of the differences between beauty and cuteness in peripheral vision. Second, participants could judge facial beauty and cuteness of the blurred faces as well as the original ones, regardless of the participant’s gender. This also suggests that image blur is not the primary cause of lower cuteness ratings in the periphery.

## General Discussion

### Judging Beauty and Cuteness in Peripheral Vision

In Experiment 1, we confirmed that beauty is detectable in both central and peripheral vision (Guo et al., 2011), while it also revealed that central vision is more suitable for judging cuteness. However, judging cuteness is more difficult in peripheral vision, especially for male participants.

In Experiment 2, we showed that this difficulty in judging cuteness did not vary with the level of blurred faces at each eccentricity. In addition, no gender difference was found. The procedural difference of presentation method between Experiment 1 and 2 (comparing two faces vs. rating single face) might affect the results. However, significant rank correlations between Experiment 1 (self-paced judgment) and Experiment 2 (each level of blurring) were found for judging both beauty and cuteness regardless of the participant’s gender (*p*s < 0.001). Therefore, it is hardly to say that the difference of stimulus presentation affect the judgments. Another important feature of peripheral vision was the weaker response to color, but this was not relevant since we used grayscale images. Therefore, the difficulty in judging cuteness in peripheral vision and the related gender difference cannot be explained by the image properties. For example, a simple hypothesis that cuteness depends on higher spatial frequency information more than beauty should be rejected. Beauty reflects averageness and symmetry (Rhodes, 2006). These sets of information might be readily available in peripheral vision as beauty judgment was not much affected. Since the high-beauty faces were almost the same as the high-cuteness ones, no difference should have been observed between beauty and cuteness if the participants had relied upon the same accessible arrangement of facial features. The difference between beauty and cuteness found in this study, accordingly, indicates that beauty and cuteness judgment should rely on different features, and that cuteness relies on features that are not accessible in peripheral vision.

Beauty was detectable in peripheral vision as well as in central vision, regardless of the participant’s gender. Conversely, cuteness was more difficult to detect in peripheral vision than in central vision, which was more pronounced in the male participants. While the underlying mechanisms are still open for further investigation, we can also understand these differences from a functional perspective. Beauty consists of averageness and symmetry, which function as indices of one’s state of health (Rhodes et al., 2001) and one’s quality of genes (Thornhill and Moller, 1997) for mate selection. Therefore, it is ecologically adaptive to first find a beautiful face in peripheral vision and then direct attention to the person. In fact, it has been reported that greater attention is directed toward a more attractive face (Shimojo et al., 2003; Leder et al., 2010). On the other hand, cuteness is evolutionally related to caregiving behaviors (Lorenz, 1943). Therefore, receiving cuteness may make the receiver observe carefully and concentrate on the cute object. This assumption is supported by the finding that cuteness can improve the performance of certain tasks that need attention (Nittono et al., 2012). However, averted attention may cause careless behaviors of the caregiver. Therefore, central vision is essential for cuteness, while judging cuteness in peripheral vision may be less important.

### Gender and Cultural Effects on Cuteness

Japanese people tend to confuse being beautiful (*utsukushii*) with being cute (*kawaii*; Daibo, 2007). In this study, the high-beauty and the high-cuteness groups consisted of almost the same facial images, which suggest that beauty and cuteness were combined by the Japanese participants to some extent. Conversely, judgment of cuteness was significantly affected by peripheral viewing, whereas that of beauty was not, thus indicating that the Japanese participants did not completely confuse the two aspects. The effect of eccentricity on cuteness judgment was particularly observed in the male participants. The possible reasons for this gender difference are as follows.

First, we used only female faces, which might have led to the asymmetric results. However, there is no straightforward reason to assume that people are more sensitive to cuteness of the same gender given that the female participants performed better. If the males had performed better in beauty judgment, then we could have argued that detecting beauty of the opposite gender quickly is advantageous in terms of mate selection. There was actually a slight tendency in which the males were better at judging beauty at 10° eccentricity than the females (**Figure 3**), and this was statistically supported by the difference in accuracy rates between 5° and 10°. However, the result that the males performed worse than females in cuteness judgment rejects the mate-selection-based explanation that males somewhat confused beauty with cuteness.

Second, it has been reported that females are more sensitive in perceiving cuteness due to female hormones (Sprengelmeyer et al., 2009). Our results suggest that females may have wider field of view in regard to cuteness judgment even though it becomes more difficult to perceive cuteness in peripheral vision. This may reflect the level of female hormones or it may be related to the general tendency that females play a more important role in caregiving behaviors. However, further investigations are needed for these suggestions.

## Conclusion

Our results showed that judging beauty is invariant in peripheral and central vision, while judging cuteness is degraded in peripheral vision. In addition, it was more difficult for the male participants to judge cuteness in peripheral vision, thus suggesting that gender differences can have a certain effect when judging cuteness. Finally, lower resolution in peripheral vision should not be the main cause of the tendency for cuteness (as described earlier) and central vision might be essential for judging cuteness while judging beauty could be detected more widely in peripheral vision. These results might be related to the functional difference between beauty and cuteness.

## Conflict of Interest Statement

The authors declare that the research was conducted in the absence of any commercial or financial relationships that could be construed as a potential conflict of interest
